# Epoxy Composites Modified with Functionalized Aluminosilicate Microspheres from Thermal Power Plant Ash: Complex Improvements in the Mechanical and Thermal Properties

**DOI:** 10.3390/polym17121666

**Published:** 2025-06-16

**Authors:** Anton Mostovoy, Andrey Shcherbakov, Gulbanu Serikbayeva, Marina Lopukhova, Victoria Svitkina, Zamzagul Shanina, Amirbek Bekeshev

**Affiliations:** 1 Laboratory of Modern Methods of Research of Functional Materials and Systems, Yuri Gagarin State Technical University of Saratov, Polytechnichskaya St., 77, 410054 Saratov, Russia; 2 Laboratory of Support and Maintenance of the Educational Process, Yuri Gagarin State Technical University of Saratov, Polytechnichskaya St., 77, 410054 Saratov, Russia; shcherbakovas@techn.sstu.ru; 3 Department of Physics, K. Zhubanov Aktobe Regional State University, Aliya Moldagulova Avenue 34, Aktobe 030000, Kazakhstan; gserikbayeva@zhubanov.edu.kz (G.S.); zshanina@zhubanov.edu.kz (Z.S.); 4 Department of Economics and Humanitarian Sciences, Yuri Gagarin State Technical University of Saratov, Polytechnichskaya St., 77, 410054 Saratov, Russia; lopuhovami@techn.sstu.ru; 5 Department of Technology and Equipment for Chemical, Oil and Gas and Food Industries, Yuri Gagarin State Technical University of Saratov, Polytechnichskaya St., 77, 410054 Saratov, Russia; lab.205@techn.sstu.ru; 6 Laboratory of Polymer Composites, K. Zhubanov Aktobe Regional State University, Aliya Moldagulova Avenue 34, Aktobe 030000, Kazakhstan

**Keywords:** epoxy composites, aluminosilicate microspheres, functionalization, mechanical properties, thermal stability, industrial waste

## Abstract

In this paper, the effect of aluminosilicate microspheres (ASMs) from thermal power plant (TPP) ash on the properties of epoxy composites was studied. A method for modifying the ASMs’ surface using aminoacetic acid was developed to improve the adhesion at the polymer–filler interface. Complex analysis methods, including scanning electron microscopy, infrared spectroscopy, a thermogravimetric analysis, DSC, and DMA, showed that adding the optimal amount of ASMs significantly improved the physical and mechanical properties of the composites: the flexural strength increased by 112%, the elastic modulus by 198%, and the impact strength by 50%. Functionalization of the ASMs enhances their interaction with the matrix, providing the composites with the best strength and thermal stability indicators among the studied materials. The study of the curing kinetics showed the initiating effect of functionalized ASMs on the curing process of epoxy compositions, associated with the presence of active amino groups on the surface of the particles. The resulting composites demonstrate potential for application in structural and fire-resistant materials; have high-deformation and -strength characteristics; and facilitate the disposal of industrial waste.

## 1. Introduction

Industries such as building material production, energy, agriculture, metallurgy, mining, etc., as a rule, produce by-products that are harmful to the environment. Recycling production waste is a complex and expensive process, which often leads to the accumulation of waste on the territory of the production complex. Although the waste generated is hazardous, it can be successfully used in the production of polymer composite materials; its use primarily serves the purpose of recycling industrial waste and reducing the cost of the finished products [1,2].

One of the most suitable polymers for recycling industrial waste is epoxy resin. Epoxy resins have proven themselves in various industries, and there are a sufficient number of studies showing that the use of industrial waste to modify epoxy resins makes it possible not only to improve their environmental stability but also to improve the properties of the resulting composites. Agnieszka Chowaniec et al. [3] modified the epoxy coating for a cement base with glass powder. It was found that the addition of this filler to an amount of 7.4–35.9% of the epoxy resin’s mass had a positive effect on its tear strength. According to the authors, this phenomenon was associated with the filling of pores at the phase boundary with glass particles, which led to an increase in the adhesion of the coating. In the work [4], red mud (a by-product of aluminum production) was used to modify an epoxy composite reinforced with coconut sheath (CS) fiber. By adding 20 wt.% red mud into the matrix in combination with chemically treating the fibers’ surface, the authors managed to obtain the optimal properties in the composite, which led to an increase in its flexural and tensile strength by 104% and 208%, respectively. At the same time, a decrease in the water absorption of the modified composites was noted due to a decrease in the contact area of the fibers with water molecules. The use of blast-furnace dust [5] as a modifier in combination with quartz sand made it possible to obtain artificial stone with high-deformation and -strength characteristics. The flexural strength of such a composite was 32 MPa relative to the 25 MPa of the pristine matrix, while this composite, when studied using the Amsler wear test method, showed an abrasion of 2.09 with a test duration of 1000 m, which would potentially allow it to be used as a road covering for medium traffic.

A significant problem is the considerable amount of plastic waste. Yuanyuan Wang et al. [6] proposed obtaining CNTs from polypropylene pyrolysis waste through chemical vapor deposition (CVD) in a two-stage fixed-bed reactor. The obtained CNTs were added into an epoxy matrix to amounts of 0.1 to 6 wt.%, the elastic modulus indices increased with an increase in the amount of CNTs, and the optimal strength indices were obtained by adding 2 wt.%.

In order to reduce the production costs of materials, with constant growth of the prices for minerals, the use of waste from the thermal power complex becomes relevant. In developed countries, constant work is being undertaken to improve the technologies for involving the relevant waste in production circulation [7,8,9]. The most valuable components of fly ash are aluminosilicate microspheres (ASMs). The formation of microspheres in fly ash is a complex multi-stage process. Microspheres are generated from mineral particles capable of forming eutectic mixtures and glass phases at temperatures above 1200 °C. Such particles include aluminosilicate clay minerals and hydromicas, together with minerals with an increased SiO_2_ content, which include quartz and feldspars. Such particles contain, at the impurity level, approximately 1–3% gas-releasing substances, as well as crystallization water. Under high-temperature exposure, when the particles reach a viscous-flowing state, this leads to the formation of hollow, monocellular, spherical particles—microspheres [10,11,12].

Microspheres obtained from TPP ash, with a yield of about 2.5 kg per ton of coal burnt, are analogous to the glass microspheres obtained through industrial methods, but their cost is several times lower. This is explained by the technological nature of the process of extracting microspheres from TPP ash. When mixing ash with water, the microspheres float to the surface, as a result of natural flotation, where they are extracted through special technical means and then dried in the laboratory under normal conditions [13,14].

The use of solid fuel combustion waste not only saves material resources but also solves the problem of waste disposal in the thermal power complex. Therefore, it is necessary to develop new materials to increase the utilization of ash dumps [15,16].

The aim of this work is modification of the formulation of epoxy resins, directed at improving their physicochemical and mechanical properties. For this purpose, the use of aluminosilicate microspheres, obtained as multi-tonnage waste from TPPs, as a highly effective filler is proposed. Both pristine aluminosilicate microspheres and their functionalized forms modified using aminoacetic acid are studied. This approach allows us not only to improve the performance characteristics of epoxy compositions but also promotes the recycling of industrial waste, which is relevant to the principles of sustainable development and resource conservation.

The scientific novelty of this work consists of establishing the influence of chemically modified aluminosilicate microspheres on the structure and properties of the epoxy matrix at different structural levels. For the first time, it has been shown that the thin interphase layer formed during modification plays a key role in the formation of the strength characteristics of the composite, ensuring effective load transfer and crack suppression. We identified the critical filler volumes at which transitions from dispersed reinforcement to the formation of a rigid framework were realized. The thermo-kinetic features of curing, caused by the reactive activity of the functionalized surfaces of the microspheres, were also established.

## 2. Materials and Methods

### 2.1. The Materials and Reagents

The following materials were used in this study: epoxy resin ED-20 (epoxy group content: 20–22%; viscosity: 16–20 Pa·s at 25 °C; density: 1.16–1.20 g/cm^3^; purity: ≥99%) and polyethylenepolyamine (PEPA) hardener (primary amine content: 28–32%; density: 0.97–1.02 g/cm^3^; purity: ≥98%), both supplied by CHIMEX Limited (St. Petersburg, Russia). These components were used as received without further purification. Trichloroethyl phosphate (TCEP) with a purity of 95–99%, which is an orthophosphoric acid and an ethylene chlorohydrin ester, produced by Xuancheng City Trooyawn Refined Chemical Industry Co. (Xuancheng, China), was used as a plasticizer and a fire retardant. The TCEP molecules contained phosphorus (10.3–11.3 wt.%) and chlorine (36.3–37.5 wt.%). Aluminosilicate microspheres (ASMs) isolated from fly ash from the Dzerzhinskaya TPP (Dzerzhinsky, Moscow Region, Russia) were used as filler, with a yield of about 2.5 kg per ton of coal burnt. Surface functionalization of the ASMs was carried out using aminoacetic acid (VitaReaktiv LLC, Dzerzhinsk, Russia).

### 2.2. Functionalization of the ASMs

Functionalization of the ASMs’ surfaces was performed in a 5% aminoacetic acid solution. This process included preliminary ultrasonic treatment for 15 min (frequency: 22 ± 2 kHz; power: 400 W) to ensure a uniform distribution of the microspheres, after which point the mixture was refluxed at 80 °C for 12 h under stirring at 100 rpm. The resulting suspension was centrifuged, washed twice with distilled water, and dried at 80 °C for 5 h [17].

### 2.3. Сharacterization of the ASMs

The morphology of the microspheres was studied through scanning electron microscopy using a Tescan MIRA 3 LMH microscope. The particles’ size distribution was determined through laser diffraction using a Fritsch Analysette-22 Nanotech instrument (measurement range: 0.01–1000 μm; dispersion medium: water). Infrared Fourier spectroscopy (Shimadzu IRTracer 100, Kyoto, Japan) and atomic emission spectroscopy with inductively coupled plasma using an Optima 4300 instrument (Perkin-Elmer, Waltham, MA, USA) were used to evaluate the functional groups and elemental composition. The bulk density was measured using a PT-TD200 PHARMA TEST device, and the true density was measured using an ACCUPYC 1330 pycnometer. An X-ray phase analysis was performed using an ARL X’tra diffractometer with CuKα radiation (2θ angle from 5 to 60°). Interpretation of the data was carried out using the ICDD database (PDF-2) and Crystallographic Search Match version 3.1.0.2.b.

### 2.4. Preparation of the Epoxy Composites

A probe disperser (frequency: 22 ± 2 kHz; power: 400 W) was used to disperse the microspheres. A mixture containing 100 parts by mass of epoxy resin ED 20 and 40 parts by mass of TCEP was processed for 60 min. After the dispersion of the composition had been completed, 15 parts by mass of the PEPA hardener was added, after which mixing was carried out at 100 rpm for 3 min. The resulting mixture was poured into silicone molds and left to cure for 72 h.

### 2.5. Composite Testing

For tensile testing, “dog bone” samples with a thickness of 4 mm and a width of 10 mm were used, and the length of the working zone was 50 mm. For the bending tests, rectangular samples with a thickness of 4 mm and a width of 10 mm were used, and the length of the working zone was 80 mm. For impact testing, rectangular samples with a width of 15 mm, a thickness of 10 mm, and a length of 120 mm were used.

The tensile and bending strength were determined using a WDW 5E testing machine (Time Group Inc., Beijing, China) at crosshead speeds of 5 mm/min (tension) and 50 mm/min (bending), in accordance with the standards [18,19]. The impact strength was determined using a LCT-50D device manufactured by Beijing United Test Co., Ltd., Beijing, China, in accordance with the standard [20].

All mechanical tests were carried out for batches of five samples. Before testing, the samples were kept for at least 72 h at 25 ± 1 °C and then subjected to heat treatment in air: for 6 h at 90 °C and 6 h at 120 °C. The results of the tests to determine the strength indicators were averaged, and the spread of values did not exceed 5%.

The heat resistance of the composites was assessed using the Vicat method (the B50 method, a 50 N load, a 50 °C/h heating rate). The curing kinetics were studied using differential scanning calorimetry (DSC) on a DTAS 1300 device (Samara, Russia) using 20 mg samples heated to 400 °C at a rate of 8 °C/min in air. A thermogravimetric analysis (TGA) was performed using a Q 1500D derivatograph (MOM, Budapest, Hungary) by heating 100 mg samples to 800 °C (rate of 10 °C/min) in air, recording the change in mass with an accuracy of ±1%.

Thus, the above set of methods and careful preparation of the samples ensured reproducible results in assessing the mechanical and thermal properties of epoxy composites with aluminosilicate microspheres.

## 3. Results and Discussion

The chemical composition of the ASMs was analyzed using inductively coupled plasma atomic emission spectroscopy on an Optima-4300 device from Perkin-Elmer, as well as infrared spectroscopy on an IRTracer-100 device from Shimadzu. These studies revealed that the main composition as assessed using AFM included oxides of silicon, aluminum, iron, calcium, magnesium, sodium, potassium, and titanium, as shown in Table 1. The presence of these oxides testifies to the complex multicomponent composition of the ASMs from fly ash, which is typical of coal combustion products. At the same time, the combination of these components in AFM confirms their environmental safety, making them suitable for the use as a filler in composite materials.

**Figure 1:**
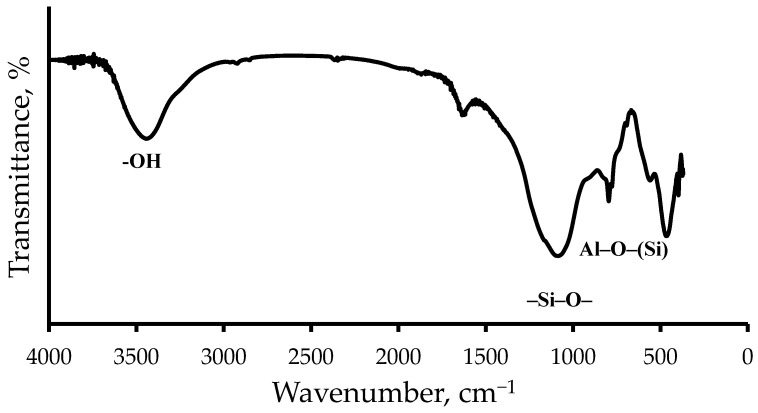
The IR spectrum of the ASMs.

The IR spectra of the ASMs, shown in Figure 1, show characteristic peaks that reflect their chemical structure. The peaks at 1090 and 796 cm^−1^ correspond to vibrations of the Si–O and Al–O–(Si) bonds in the tetrahedra that form the aluminosilicon oxygen framework. These absorption bands confirm the presence of a silicate and aluminosilicate structure in the ASMs’ composition. In addition, the spectrum contains peaks in the region of 3754–3445 cm^−1^ which are associated with vibrations of the O–H bonds. The peak at 3754 cm^−1^ corresponds to isolated Si-OH groups, and the band in the region of 3445 cm^−1^ indicates the presence of hydrogen-bonded Si-OH groups [21,22]. The presence of these peaks indicates the presence of hydroxyl groups on the surface of the ASM particles, which can affect their reactivity and interaction with other components of the composition. The IR spectroscopy data obtained confirm the complex structure of the ASMs and their chemical composition, which are consistent with the results of atomic emission spectroscopy.

**Figure 2:**
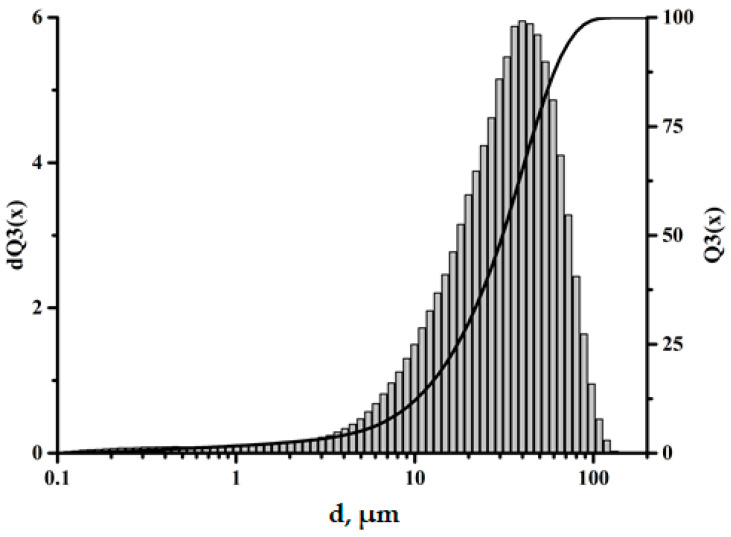
Fractional composition of the ASMs.

**Figure 3:**
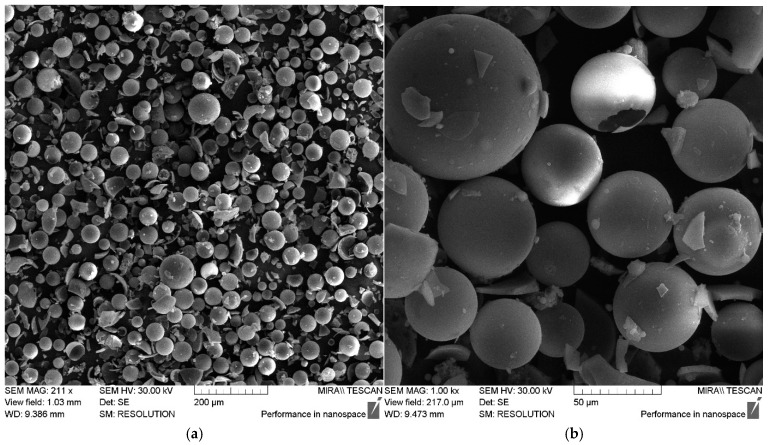
SEM images of ASMs: (**a**) 211×; (**b**) 1.00 k×.

The size and distribution of filler particles significantly affect the characteristics of polymer composites [23,24]. The fractional composition of the microspheres used in this work can be characterized as monomodal, with the particle sizes varying from 0.5 to 100 μm. The largest volume fraction is accounted for by particles 30–60 μm in size, as shown in Figure 2. The data obtained using scanning electron microscopy (SEM) also confirm this size range, as shown in Figure 3.

According to the SEM data, as shown in Figure 3, the ASM particles have a shape close to spherical and a smooth outer surface.

The bulk and true density of the ASMs were also determined, which were 0.30–0.45 g/cm^3^ and 0.35–0.50 g/cm^3^, respectively.

The content of moisture and volatile products in the ASMs is 0.2 ± 0.01%, which indicates that drying is required when polymer composite materials are obtained since increased moisture content in the filler will primarily affect the nature of the interaction of the polymer matrix and filler and will also lead to the formation of pores and voids at the polymer–filler interface and, as a consequence, could cause a decrease in the performance properties of polymer composites. The removal of moisture and volatile products was carried out at a standard temperature of 105 °C.

Studies of the granulometric and chemical composition of the ASMs show that they can be used as an inexpensive filler which should improve the physicochemical and mechanical properties of polymer composite materials.

The main advantages of spherical fillers are their small surface-area-to-volume ratio, which promotes low adsorption of the binder; their perfect shape, which ensures good wetting of the particles and a uniform distribution of stresses in the material; the possibility of surface modification; and the high resistance of polymer composite materials filled with spherical fillers to stretching, compression, and water resistance, as well as the high heat resistance of ASMs (melting point: 1400–1500 °C). Moreover, the chemical composition of ASMs ensures their high resistance to acids and alkalis, and they are pH-neutral and do not affect the chemical composition or reactions of materials or products in which they are used [25,26,27].

The polymer matrix was formed on the basis of a composite composition including the following components: 100 parts by mass of ED-20 epoxy resin; 40 parts by mass of TCEP; 15 parts by mass of the hardener—PEPA.

TCEP in this composition performs two key functions: a plasticizer that improves the mechanical properties of the material and a fire retardant that increases the fire resistance of the composite due to the presence of combustion inhibitors, phosphorus and chlorine, which reduce the flammability in both the gas and condensed phases. When heated, TCEP releases non-flammable gases (hydrogen chloride and phosphorus-containing compounds), which dilute flammable vapors and slow down the combustion process. In addition, phosphorus has a structuring effect, increasing the yield of carbonized structures that are a physical barrier to the interdiffusion of flammable gases and oxygen into the combustion zone [28]. The introduction of TCEP into the epoxy matrix leads to a significant improvement in a number of key physical, mechanical, and fire protection characteristics: the bending stress value increases from 40 to 53 MPa, which indicates an increase in the strength of the material; the impact resistance increases from 3 to 8 kJ/m^2^, indicating improved resistance to dynamic impacts; and the flammability index (oxygen index (LOI)) increases from 19 to 27% by volume, which ensures the transition of the material to the class of hardly combustible. In addition, TCEP reduces the rate of flame propagation and reduces smoke formation, which further improves the fire safety of the composite when used in conditions of elevated temperatures or risks of fire [29].

In this study, the effect of the ASMs on the physical and mechanical properties of the epoxy composites was studied when they were used as a modifying additive and a filler. ASMs were added into the epoxy composition in a range from 0.05 to 0.5 parts by mass as a modifying additive and from 10 to 60 parts by mass as a filler. The results of these studies made it possible to determine the optimal content of ASMs which ensured the maximum improvement in the properties of the composites.

Based on the data presented in Table 2, it was established that the most rational content of ASMs in epoxy composites is 0.1 parts by mass as a modifying additive and 50 parts by mass as a filler. At such concentrations, the maximum physical and mechanical properties of the composites are achieved. The addition of ASMs to an amount of more than 50 parts by mass leads to a significant increase in the viscosity of the epoxy composition, which complicates its processing and molding. Moreover, when this content is exceeded, a sharp decrease in the strength characteristics of the material is observed, which makes the use of ASMs at such amounts irrational; see Table 2.

Low additions of aluminosilicate microspheres (ASMs) into the epoxy composite significantly improve the complex of physical and mechanical characteristics of the material. According to the data presented in Table 2, the following changes in the properties of the composite are observed compared to those of the plasticized epoxy composition that does not contain ASMs: the bending and tensile strength increase by 112 and 61%, respectively; the modulus of elasticity in bending and stretching increases by 40 and 14%, respectively, which indicates an increase in the rigidity of the material, making it more resistant to deformation; the compressive strength increases by 33%, confirming that ASMs effectively distribute the load in the material, preventing the formation of cracks and defects; and the resistance to dynamic and impact effects increases, which is expressed in an increase in the impact strength by 50%.

In terms of reducing the production cost, the addition of ASMs as a filler to the epoxy composite (50 parts by mass) is an effective solution. At the same time, an improvement in the physical and mechanical characteristics of the material is observed. In particular, the bending strength increases by 66%, the modulus of elasticity in bending increases by 198%, the tensile strength increases by 17%, and the modulus of elasticity in stretching increases by 95%. Moreover, an increase in the impact strength of 12.5% is noted compared to that of the unfilled plasticized epoxy composite; see Table 2.

In terms of the energy concept, the strengthening of the epoxy composites filled with ASMs is explained by the increase in the energy required to destroy the material. This increase is due to the additional energy costs associated with the formation of a new surface when a crack passes through the material. In particular, the crack is forced to flow around the filler particles, which leads to an extension of the crack front and an increase in the energy spent on its propagation [30,31,32,33]. In addition, the filler particles affect the processes of the structure formation and orientation of macromolecules, which leads to the improvement in the structural organization of the material [34,35]. Thus, the presence of ASMs in the polymer matrix not only improves the structural characteristics of the material but also increases its strength and resistance to destruction due to the energy barriers created by the filler particles.

The obtained data indicate that the use of ASMs as a filler not only helps to reduce the production costs but also improves the performance properties of the composite material, making it more durable and resistant to deformation and impact loads. This makes them promising for the application of such composites in various industries that require materials with high mechanical characteristics and cost-effectiveness.

Filler functionalization is indeed an important approach to improving the properties of composite materials, especially those based on an epoxy matrix [36]. Surface modification of the filler particles allows two key problems to be solved: preventing particle aggregation and improving their interaction with the polymer matrix [37,38,39]. In this regard, the use of aminoacetic acid as a functionalizing agent for the ASMs in this study is particularly promising. Aminoacetic acid has a dual functionality due to its presence of an amino group (-NH_2_) and a carboxyl group (-COOH). These functional groups play a key role in improving the properties of the composite. The presence of the amino group ensures the formation of strong chemical bonds with the epoxy matrix, which improves the adhesion between the filler and the polymer [40]. This interaction helps to increase the mechanical strength of the composite, as it ensures more efficient transfer of the load from the matrix to the filler. In addition, the compatibility of the filler with the polymer matrix improves, which reduces the possibility of delamination and defects in the material [41].

The presence of a carboxyl group in the composition of the ASMs ensures interaction with the surfaces of the filler particles, which makes their uniform dispersion in the polymer matrix possible and prevents agglomeration of the particles, which is crucially important for achieving a homogeneous structure in the composite [42,43].

These effects make filler functionalization using aminoacetic acid a promising method for creating high-quality composite materials with improved performance characteristics.

**Figure 4:**
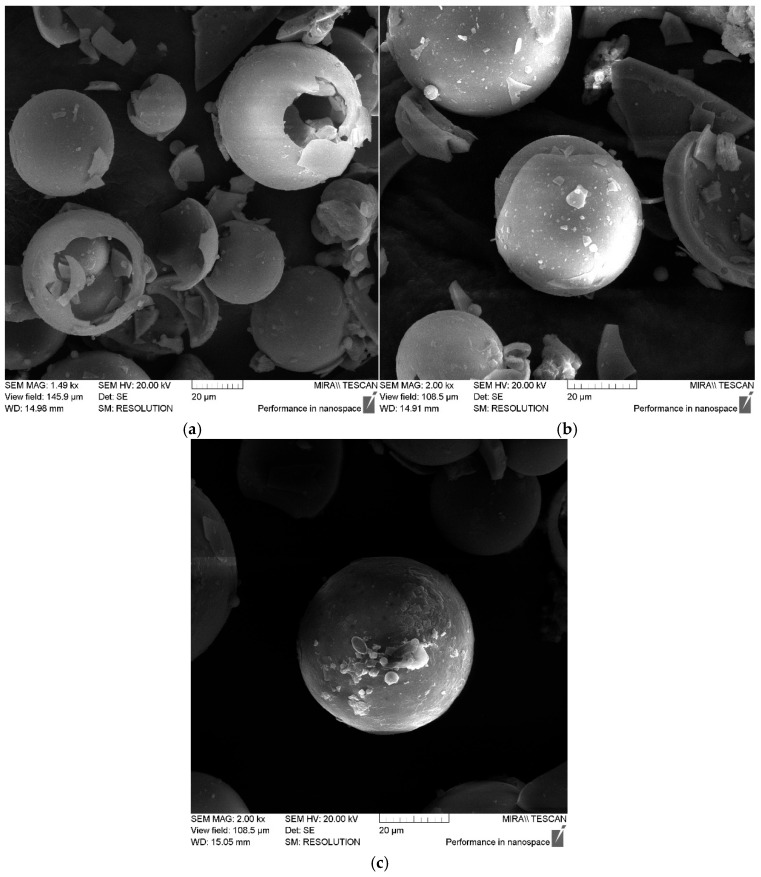
SEM images of ASMs treated with aminoacetic acid at magnifications: (**a**) 1.49 k×, (**b**) 2.00 k×, (**c**) 2.00 k×.

**Figure 5:**
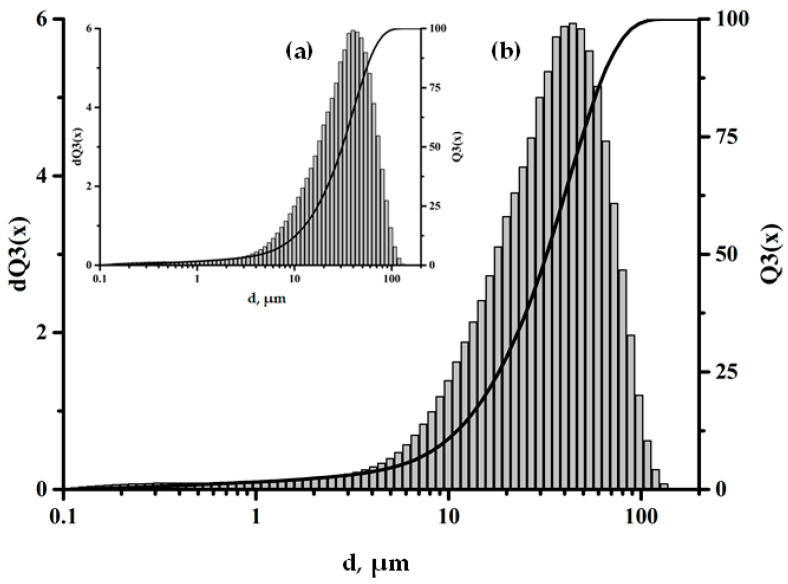
Fractional composition of the particles: (**a**) pristine ASMs; (**b**) ASMs treated with aminoacetic acid.

The SEM data confirm the uniform distribution of adsorbed amino acid molecules over the surfaces of the aluminosilicate microspheres, which indicates effective functionalization and the formation of a homogeneous modified surface layer; see Figure 4. It is important to note that the treatment of the ASM particles with aminoacetic acid does not change their fractional composition, which is confirmed by the data presented in Figure 5. This indicates that the modification process does not have a significant effect on the size or shape of the particles, preserving their original characteristics, which is important to ensuring the stability of the properties of the composite material.

**Figure 6:**
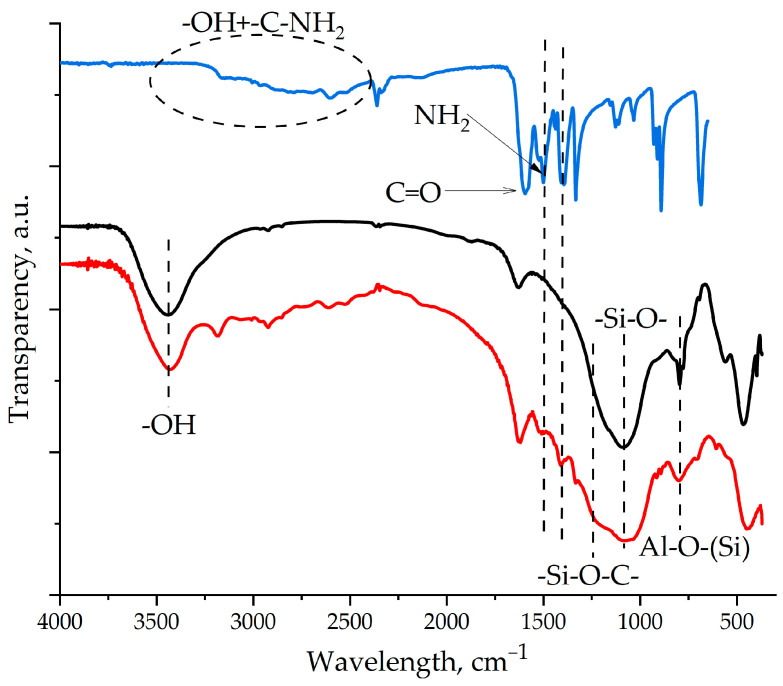
FT-IR spectroscopy of the samples: 1—aminoacetic acid; 2—pristine ASMs; 3—ASM samples treated with aminoacetic acid.

The chemical interaction of the amino groups in aminoacetic acid with the epoxy groups in the ED-20 oligomer was proven in our previous studies and presented in [44]. After treating the surfaces of the ASMs with aminoacetic acid, characteristic vibration peaks corresponding to the functional groups of aminoacetic acid are observed in the IR spectra of the modified samples, as shown in Figure 6, which confirms the presence of molecules of aminoacetic acid on the surface of the ASMs. In addition, broadening of the peak in the region of 1100–1000 cm^−1^ is noted, which is associated with the formation of the chemical bond -Si-O-C-. It is important to note that these peaks are preserved after washing the samples with water, which indicates the non-hydrolyzable nature of the -Si-O-C- bond and its resistance to water. This confirms the strength and stability of the modification. The formation of a stable chemical bond between aminoacetic acid and the ASMs shows the strong fixation of the molecules of aminoacetic acid onto the surfaces of the microspheres. Thus, the results of IR spectroscopy confirm successful chemical modification of the ASMs’ surface, which opens up prospects for their use as a functional filler in epoxy composites.

**Figure 7:**
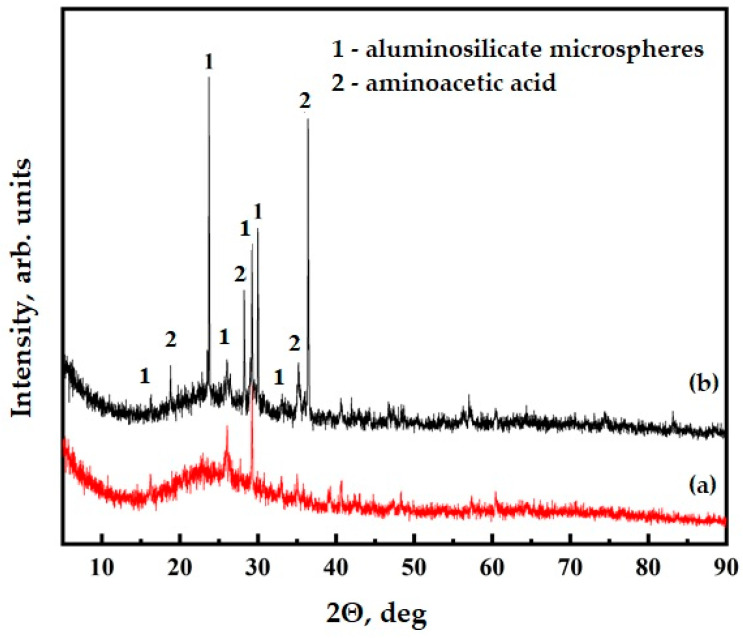
XRD data for ASM: (**a**) pristine ASMs; (**b**) ASMs after treatment with aminoacetic acid.

The presence of functional groups on the surface of the ASMs was confirmed further through the X-ray diffraction (XRD) analysis. The XRD patterns of the unmodified ASMs, illustrated in Figure 7, curve (a), display characteristic peaks at specific 2θ angles, which are typical of this material, particularly corresponding to quartz (2θ ≈ 26.6°, 20.8°, 35.0°, and 50.1°) and mullite (2θ ≈ 16.4°, 25.9°, 30.4°, and 40.8°), alongside a broad amorphous halo between 22 and 24° due to the glassy phase [45,46]. After the functionalization process, new weak peaks corresponding to crystalline aminoacetic acid were observed at approximately 19.0°, 29.8°, and 36.2°, while the original structural features of the ASMs were retained without significant shifts or intensity changes, as demonstrated in Figure 7, curve (b). The calculation of the crystallite sizes for quartz peaks (using Scherrer’s equation [47] on the peak at 26.6°) indicated no significant difference between the pristine and functionalized samples (~23 nm), confirming that the functionalization process did not negatively affect the inherent crystalline structure. Additionally, the absence of characteristic peaks for calcium carbonate (~29.4°) indirectly indicates that the amino groups more likely bonded through Si–O–C linkages than through forming carbonate species. This result provides clear evidence of successful, gentle functionalization of the ASMs’ surfaces. The preservation of the original ASM structure alongside the appearance of new peaks confirms that the functionalization process did not alter the fundamental properties of the material but effectively introduced the desired functional groups. These results emphasize the efficiency of the functionalization approach and its potential to enhance the application of this material in various composite systems.

**Figure 8:**
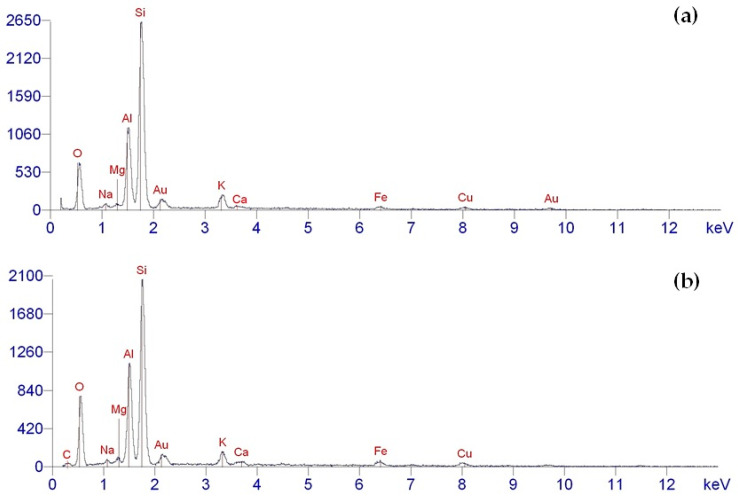
EDX data for ASM: (**a**) pristine ASMs; (**b**) ASMs after treatment with aminoacetic acid.

The elemental composition of both the pristine and aminoacetic-acid-functionalized ASM particles was analyzed using the EDX method. The results indicate that the chemical composition of the pristine ASM particles aligns perfectly with the data acquired through inductively coupled plasma atomic emission spectroscopy, as illustrated in Figure 8a. The EDX analysis of the unmodified ASMs revealed the presence of Si, Al, Fe, Mg, Ca, and K, which matches the expected chemical composition of the ASMs. Moreover, oxygen was also identified, indicating that these elements exist in oxide form and also correspond to the existence of hydroxyl groups on the particles’ surfaces, as shown in Figure 8a.

The elemental composition of he ASMs modified with aminoacetic acid is illustrated in Figure 8b. The analysis of the acquired EDX results reveals the existence of elements such as carbon (C) and oxygen (O). Notably, the intensity of these peaks is considerably greater compared to that of the unmodified ASMs, which can be explained by the attachment of aminoacetic acid molecules to the ASM particles’ surfaces. This alteration in the modified ASM particle spectra suggests that the modification process was effective, resulting in the addition of new functional groups that could take part in chemical interactions with the polymer matrix.

These findings provide strong evidence for the successful functionalization of ASM particles with aminoacetic acid, which enhances their potential for use in composite materials by improving the interfacial interactions with the polymer matrix.

These studies showed that treatment of the ASMs with aminoacetic acid as a functionalizing agent significantly improves the interaction of the filler with the polymer matrix. This treatment effectively reduces the surface energy at the interface between the polymer and the filler, which contributes to an increase in adhesive properties [40,48]. As a result, the deformation and strength characteristics of the composite material are significantly improved.

A detailed study was conducted to assess the effect of modifying the ASMs with aminoacetic acid on the properties of the epoxy’s composition. The experimental results demonstrated a significant improvement in all of the physical and mechanical properties of the composite studied. The strength and deformation characteristics increased by 12–40% compared to those of the composite containing the untreated ASMs; see Table 2. These data confirm the effectiveness of using modified fillers to create composite materials with improved performance properties.

**Figure 9:**
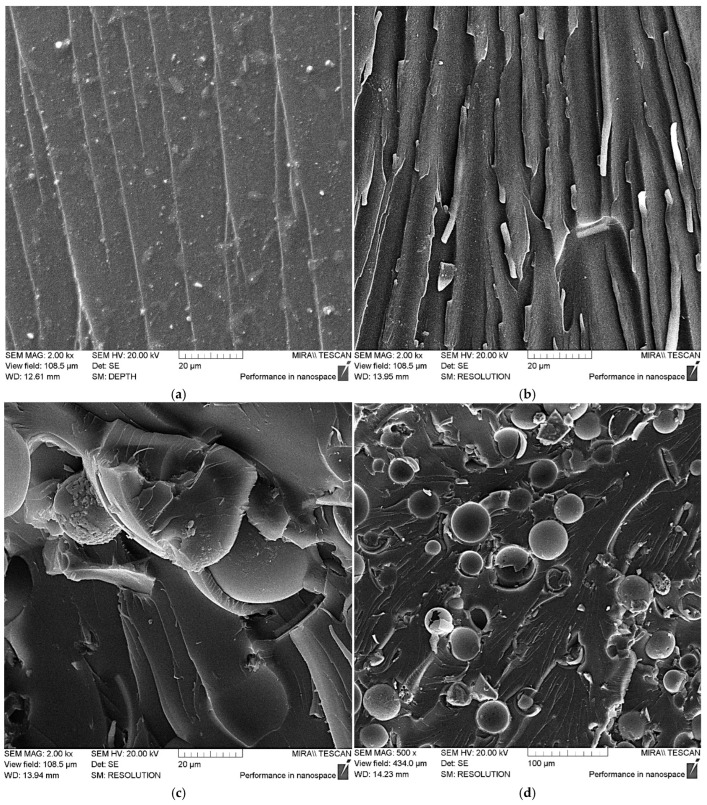

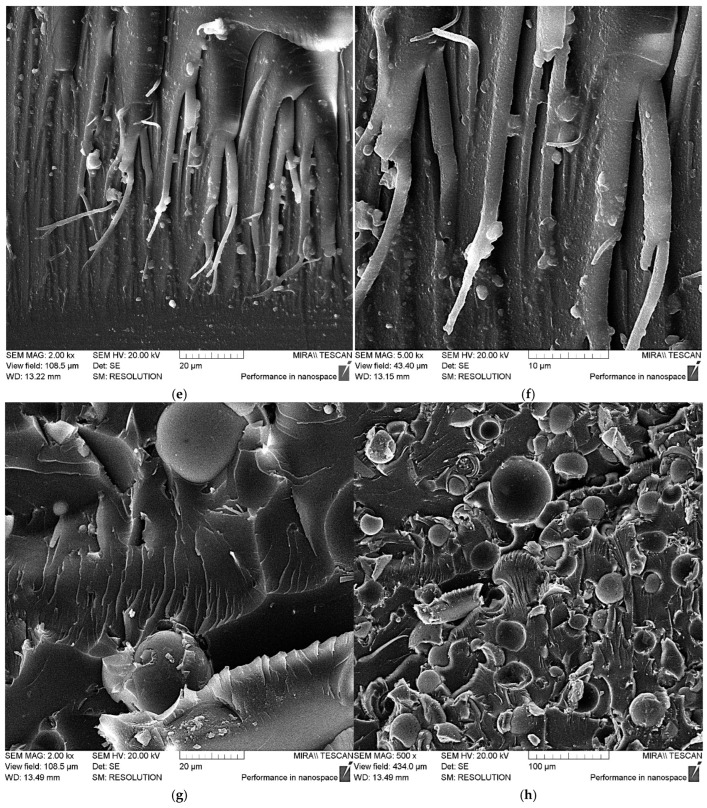
SEM data for epoxy composite samples, parts by mass: (**a**) 100 ED-20 + 40 TCEP + 15 PEPA; (**b**) 100 ED-20 + 40 TCEP + 0.1 ASM + 15 PEPA; (**c**,**d**) 100 ED-20 + 40 TCEP + 50 ASM + 15 PEPA; (**e**,**f**) 100 ED-20 + 40 TCEP + 0.1 ASM_(aminoacetic acid)_ + 15 PEPA; (**g**,**h**) 100 ED-20 + 40 TCEP + 50 ASM_(aminoacetic acid)_ + 15 PEPA.

Figure 9a illustrates that the unmodified epoxy matrix exhibits a relatively smooth chip surface, with microcrack propagation occurring in parallel, suggesting that minimal energy is necessary to fracture the matrix. When ASMs are added into the matrix, the chip’s characteristics shift to a more rigid form, as depicted in Figure 9b–d. This results in an increase in both the number and depth of the defects, indicating that additional energy is needed to initiate and grow microcracks that weaken the composite.

It should be noted that the addition of ASMs into the epoxy composite leads to some strengthening, but this strengthening effect is low, which is explained by the insufficiently uniform distribution of the ASM particles and their low capacity to adhere to the polymer matrix, which is clearly visible in the SEM data for the composites containing 50 parts by mass of the pristine ASMs; see Figure 9d.

In addition, the SEM results for the composites containing the ASMs treated with aminoacetic acid show that the modified ASM particles are uniformly distributed in the epoxy composite.

The observed plastic deformations on the chip ridges, resulting from intense stretching of the composite, support the idea that the aminoacetic-acid-treated ASMs function as a solid-state hardener, likely due to the creation of a strong crosslinking network surrounding the ASM particles and the epoxy oligomers. Hence, the overall enhancement in strength is attributed to the increased energy required for microcrack formation and advancement [49,50].

**Figure 10:**
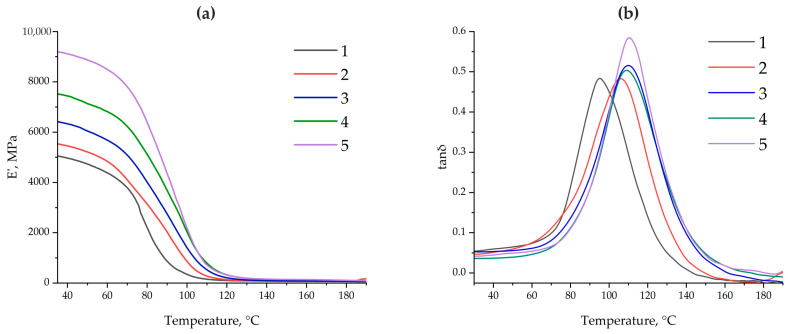
The dependence of the dynamic modulus of elasticity E′ (**a**) and the mechanical loss factor tanδ (**b**) on temperature for epoxy composites: 1—100 ED-20 + 40 TCEP + 15PEPA; 2—100 ED-20 + 40 TCEP + 0.1 ASM + 15 PEPA; 3—100 ED-20 + 40 TCEP + 0.1 ASM_(aminoacetic acid)_ + 15PEPA; 4—100 ED-20 + 40 TCEP +50 ASM + 15 PEPA; 5—100 ED-20 + 40 TCEP + 50 ASM_(aminoacetic acid)_ + 15 PEPA.

Figure 10 shows the dependence of the dynamic elastic modulus (E′) and the mechanical loss factor (tanδ) on temperature for epoxy composites containing different amounts (0.1 and 50 parts by mass) of ASMs, as well as their functionalized analogs. It is evident that adding ASMs (both non-functionalized and functionalized) increases the modulus E′ in the glassy state (at temperatures below Tg) compared to that in the original system without a filler (sample no. 1). Moreover, increasing the filler content to 50 parts by mass (samples no. 4 and no. 5) leads to an even more pronounced increase in the modulus; see Table 3. It is noted that the functionalized microspheres (in compositions no. 3 and no. 5) contribute to the formation of a more rigid structure than that in equivalent systems containing the same volumes of non-functionalized ASMs (no. 2 and no. 4). This indicates the more effective interaction of the filler with the epoxy matrix, probably due to the formation of additional interphase bonds between the aminoacetic groups on the ASMs’ surfaces and the reactive groups of the epoxy oligomer.

The glass transition temperature Tg was determined as the maximum of the main peak using the tanδ curves; see Figure 10b. In all modified compositions, Tg increases relative to that in the pristine composite (curve No. 1), and the functionalization of the ASMs additionally increases Tg. Thus, in samples with 50 parts by mass of the functionalized filler, the maximum point on the tanδ curve is shifted 2.9 °C higher than that in the equivalent composition with non-functionalized ASMs; see Table 3. A similar trend is observed with a smaller amount of ASMs (0.1 parts by mass): with the same mass of filler, on adding the functionalized ASMs, Tg in the composite is 4.1 °C higher than that in the composite containing the pristine ASMs. This increase in Tg can be explained by the formation of a more rigid interphase zone and additional crosslinks formed during the interaction of the aminoacetic groups with reactive sections of the epoxy matrix. As a result, greater thermal energy is required to implement segmental mobility and relax the polymer, which manifests itself as an increase in the glass transition temperature [51,52].

At temperatures exceeding Tg, the dynamic modulus E′ in all of the systems considered decreases to the same order of magnitude and actually “converges” to close values. This result corresponds to the typical behavior of thermosetting polymers in a highly elastic state, when the crosslinking density of the base epoxy matrix plays a decisive role [53]. Since the ratio “resin:plasticizer:hardener” (ED-20:TCEP:PEPA) remains constant in all compositions, the global density of covalent crosslinks remains approximately the same. Fillers (even functionalized ones) in the rubber-like region do not form a rigid framework and have less of an effect on the elastic–mechanical properties than they would in the glassy region. Thus, the differences that appear when measuring E′ and tanδ below and near Tg are actually leveled out in the high-temperature region, where the matrix is in a mobile “rubber-like” state.

The DMA data demonstrate a direct relationship between the ASMs’ mass and the limitation of the segmental mobility of the epoxy chains. The increase in the storage modulus (E′) and the shift of Tg to higher temperatures indicate a decrease in the free movement of the macromolecules. When 0.1 parts by mass of ASMs is added to the epoxy composite, the increase is mainly due to the physical “anchoring” of the chains to the surface of the particles, while the functionalized ASMs additionally form covalent bonds with the epoxy matrix, which increases the rigidity and further limits the mobility (E′ = 6441 MPa, Tg = 109.9 °C). With an ASM content of 50 parts by mass, a dense rigid framework is formed: E′ increases to 9235 MPa and Tg to 111.9 °C, which indicates the actual “fixation” of the chains [54,55]. Thus, the mass fraction and surface functionalization of the ASMs are key factors controlling the dynamics of the chains in the epoxy matrix.

The comparative analysis of the obtained DMA data revealed a fundamentally new effect: despite the traditional opposition between rigidity and damping capacity, the functionalized ASMs provide the formation of an interfacial architecture that simultaneously promotes an increase in the elastic modulus (E′ increases) and efficient energy dissipation (tan δ increases). This effect is due to the presence of dynamic covalent bonds that involve the interphase layer in joint relaxation processes. The bonds formed at the matrix–filler interface can be broken and restored under a load, providing dissipation energy without destroying the structure [56]. Thus, for the first time, the possibility of targeted synthesis of epoxy composites with simultaneously enhanced structural rigidity and internal damping has been demonstrated, which opens up prospects for the use of such systems in vibration-loaded and shock-resistant engineering structures.

When studying the effect of the filler on the structure formation processes in crosslinked polymers, it is necessary to take into account that the curing process occurs in the presence of an active surface of the finely dispersed filler. This factor can have a significant effect on the kinetics of polymerization and the structure formation of the material [57,58]. In this regard, this work studies the effect of adding both pristine and modified ASMs into the epoxy composition on the kinetic parameters of the curing process. The analysis was carried out using a thermometric method, which allowed us to record the temperature changes during the exothermic polymerization reaction, and a differential scanning calorimetry (DSC) method, which provided information on the thermal effects, the reaction onset temperature, the degree of conversion, and other kinetic characteristics. The obtained data allow us to evaluate the effect of the filler on the curing rate, the temperature parameters of the process, and the structural features of the final material, which is important for optimizing the composition and controlling their properties.

**Figure 11:**
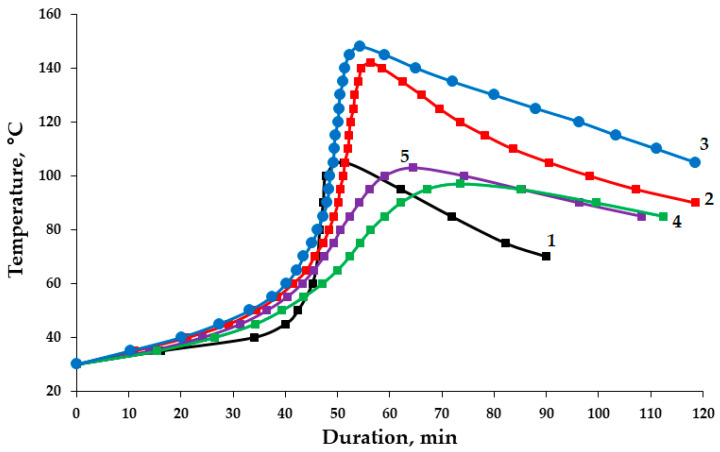
Kinetic curves for curing of the epoxy compositions: 1—100 ED-20 + 40 TCEP + 15 PEPA; 2—100 ED-20 + 40 TCEP + 0.1 ASM + 15 PEPA; 3—100 ED-20 + 40 TCEP + 0.1 ASM_(aminoacetic acid)_ + 15 PEPA; 4—100 ED-20 + 40 TCEP + 50 ASM + 15 PEPA; 5—100 ED-20 + 40 TCEP + 50 ASM_(aminoacetic acid)_ + 15 PEPA.

An analysis of the kinetic curves of the curing process of epoxy compositions containing both pristine and functionalized ASMs allows us to conclude that these additives have an initiating effect on structure formation processes; see Figure 11. The addition of ASMs treated with aminoacetic acid into the epoxy’s composition accelerates the curing process, reducing the duration of the gelation process from 40/42 to 36/40 min. This fact confirms that the functionalized ASMs contribute to the more rapid onset of the formation of the polymer network structure and reduce the duration of the curing process from 58/73 to 54/64 min, which indicates the acceleration of the polymerization processes and the formation of the final structure of the composition. An increase in the maximum curing temperature from 142/97 to 148/103 °C is noted, which is associated with an increase in the exothermic effect due to a more active polymerization reaction in the presence of functionalized ASMs, with an ASM content of 0.1/50 parts by mass, accordingly, as shown in Table 4, compared to that in the composition containing the pristine ASMs. Moreover, an increase in the degree of curing of the epoxy composites containing the functionalized ASMs has been established, which further confirms the participation of the functional groups of aminoacetic acid in the curing process; see Table 4.

**Figure 12:**
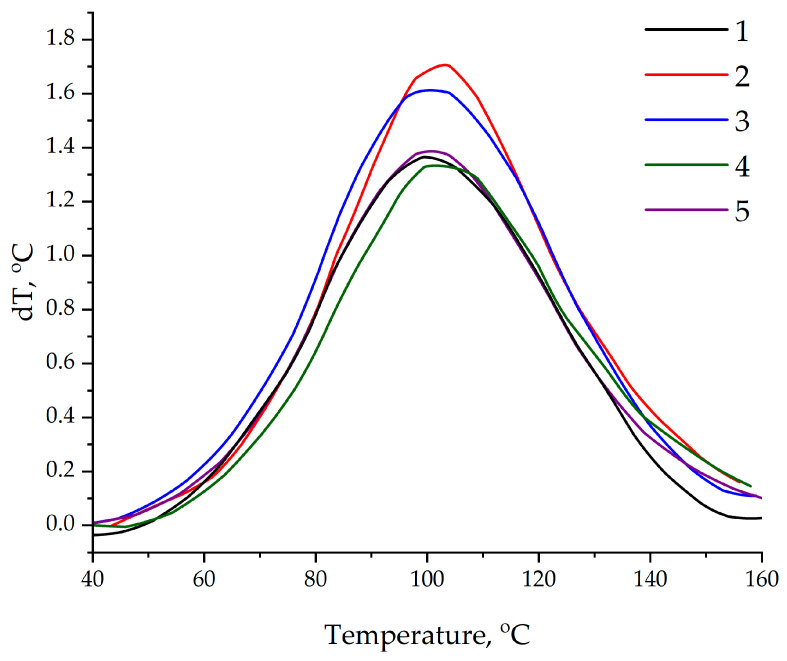
DSC data on curing of epoxy compositions: 1—100 ED-20 + 40 TCEP + 15 PEPA; 2—100 ED-20 + 40 TCEP + 0.1 ASM + 15 PEPA; 3—100 ED-20 + 40 TCEP + 0.1 ASM_(aminoacetic acid)_ + 15 PEPA; 4—100 ED-20 + 40 TCEP +50 ASM + 15 PEPA; 5—100 ED-20 + 40 TCEP + 50 ASM_(aminoacetic acid)_ + 15 PEPA.

The differential scanning calorimetry data confirmed those obtained using the thermometric method; see Figure 12. The addition of the ASMs functionalized with aminoacetic acid into the epoxy matrix increased the enthalpy of the reaction by 10.7% (from 540.6 to 598.2 J/g) and 6.6% (from 472.7 to 503.7 J/g) for compositions with an ASM content of 0.1 and 50 parts by mass, respectively, compared to that in the compositions containing the pristine ASMs. The observed increase in the thermal effect indicates an increase in the degree of crosslinking of the polymer matrix, which is due to the formation of additional covalent bonds between the functional groups of the filler and the epoxy groups of the resin [59,60]. Moreover, a decrease in the onset temperature of curing by 3.2 °C (from 46.2 to 43.0 °C) and a significant decrease by 10.6 °C (from 52.2 to 41.6 °C) were found for samples with 0.1 and 50 parts by mass of the ASMs, respectively; see Table 5. The obtained data indicate the pronounced catalytic effect of functionalized ASMs, associated with the presence of active amino groups on the surface of the particles.

The addition of both pristine and modified ASMs into the epoxy composite significantly increases the heat resistance of the material. It was found that the heat resistance index of the composite increased from 100 °C to 127–178 °C; see Table 2. It should be noted that the most pronounced improvement in heat resistance is observed when using ASMs pre-treated with aminoacetic acid. This indicates that modification of the filler’s surface plays a key role in enhancing the thermal properties of the composite, which may be due to the improved interaction at the polymer–filler interface and optimization of the material’s structure [61,62].

**Figure 13:**
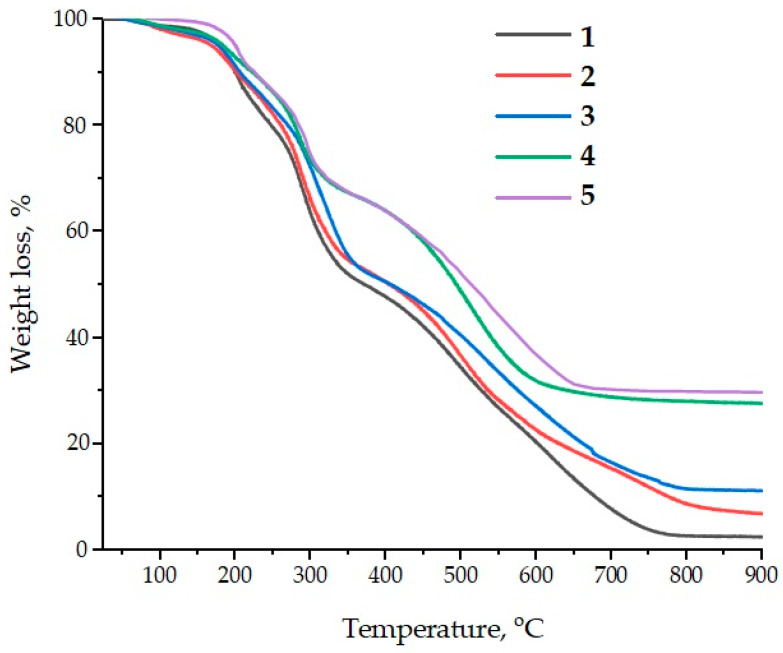
Data from the thermogravimetric analysis of the samples: 1—100 ED-20 + 40 TCEP + 15 PEPA; 2—100 ED-20 + 40 TCEP + 0.1 ASM + 15 PEPA; 3—100 ED-20 + 40 TCEP + 0.1 ASM_(aminoacetic acid)_ + 15 PEPA; 4—100 ED-20 + 40 TCEP + 50 ASM + 15 PEPA; 5—100 ED-20 + 40 TCEP + 50 ASM_(aminoacetic acid)_ + 15 PEPA.

The application of ASMs as fillers for epoxy composites is of considerable interest in terms of improving their thermal resistance and thermal stability. Both the pristine and functionalized ASMs demonstrate the ability to increase the thermal stability of epoxy materials, which is confirmed by the results of the thermogravimetric analysis (TGA); see Figure 13. In particular, an increase in the temperature characteristics of the composites in the range T_5%_–T_70%_ is observed, which indicates an improvement in their resistance to thermal decomposition.

Particular attention is paid to the ASMs treated with aminoacetic acid. Their addition into the epoxy matrix leads to a further improvement in the thermal stability of the composites, which is seen in a further increase in temperatures T_5%_–T_70%_. Moreover, a significant increase in the residual yield of carbon at high temperatures confirms the efficiency of aluminosilicate microspheres as a catalytic filler. Thus, if the carbon residue for a pure epoxy polymer is only 2.4 mass%, then for composites containing aluminosilicate microspheres, this figure increases to 6.9–29.8 wt.% at 900 °C depending on the filler concentration; see Table 6.

This increase in the residual carbon yield is due to the excellent ability of the silicon-containing filler to catalyze carbonization processes. A high residual yield is an important indicator of the thermal stability of materials at extreme temperatures, which makes ASMs a promising component for creating heat-resistant epoxy composites. Thus, the addition of aluminosilicate microspheres into the epoxy matrix not only improves its thermal stability but also helps to increase the fire resistance of the material, which opens up new possibilities for their use in high-temperature conditions.

This investigation demonstrated that structural modifications in epoxy composites significantly influence their mechanical/thermal/physicochemical properties.

Functionalization of the microspheres’ surface with amino acids provides the appearance of active amino groups capable of forming strong chemical bonds with the epoxy matrix. This leads to the formation of a stronger and more uniform “matrix–filler” interphase layer, a reduction in defects, a reduction in the probability of microsphere agglomeration, and, accordingly, a more uniform distribution of the load and more efficient load transfer between the matrix and the filler.

An analysis of the morphology of the destroyed surfaces shows that using functionalized microspheres (treated with amino acid), a more uniform distribution of particles throughout the volume of the polymer matrix, the absence of agglomerates, and tight contact between the filler particles and the epoxy phase are observed. In the destruction area, zones of plastic deformation with characteristic traces of stretching are recorded, which indicates a developed mechanism of energy-intensive destruction with enhanced interphase adhesion.

Further confirmation of the enhancement in interphase interactions was obtained within the dynamic mechanical analysis. The obtained data allowed us to confirm that unique hybrid composites were developed that combined high-rigidity and good damping properties.

Thus, this complex of morphological and thermodynamic data allows us to reliably claim that chemical modification of the ASMs’ surfaces contributes to an improvement in the interphase compatibility, structural homogeneity, and, as a consequence, a comprehensive increase in the performance characteristics of the material obtained.

Table 7 presents comparative data on the mechanical properties of various epoxy composites modified using micro-sized fillers based on industrial waste and natural raw materials. The presented compositions demonstrate the different effects of the type and amount of filler on the indicators of bending and tensile strength, elastic modulus, impact strength, and hardness. The analysis of the obtained results emphasizes the effectiveness of using ASMs modified with amino acid as a promising filler, providing high-level mechanical properties in the epoxy composites compared to those of other common waste and natural materials.

## 4. Conclusions

The conducted studies demonstrated the high efficiency of using aluminosilicate microspheres (ASMs) from TPP ash as a filler for epoxy composites. The optimal amount of ASMs is 0.1 parts by mass as a modifying additive and 50 parts by mass as a filler, which ensures the maximum improvement in the mechanical characteristics of the epoxy composites. Functionalization of the ASMs’ surfaces with aminoacetic acid leads to the formation of strong chemical bonds at the polymer–filler interface, which can be confirmed using a set of physicochemical research methods. The obtained composites demonstrate a significant improvement in a set of properties: increases in the flexural strength by 112%, elastic modulus by 198%, impact strength by 50%, the glass transition temperature by 4–16 °C, and the residual carbon yield to 29.8 wt.%. at 900 °C. The obtained data indicate the pronounced catalytic effect of functionalized ASMs on the curing process for epoxy composites, associated with the presence of active amino groups on the surfaces of the particles. The proposed approach not only allows materials with improved performance characteristics to be created but also helps solve the urgent environmental problem of recycling industrial waste from the heat and power industry. The developed epoxy composites reinforced with aluminosilicate microspheres open up broad application prospects in various fields of technology due to their combination of a low density, high strength, and thermal stability. Materials of this type can be effectively used in the aerospace industry for lightweight structural panels and heat-insulating coatings, significantly reducing their weight and energy costs. Their high impact strength makes them promising for the manufacture of protective elements for car bodies and sports equipment. In addition, their fire resistance and chemical inertness allow us to recommend these composites for use in construction, electronics, and marine engineering, where the durability and safety of structures are especially important.

## Figures and Tables

**Table 1:** Chemical composition of ASMs.

Indicator	SiO_2_	Al_2_O_3_	Fe_2_O_3_	CaO	MgO	K_2_O	Na_2_O	TiO_2_
Mass fraction %	49–51	24–26	8–9	3–4	1.4–1.6	1.6–1.8	0.9–1	0.8–1

**Table 2:** Properties of epoxy composites.

Composition, Parts by Mass, Cured with 15 Parts by Mass of PEPA	σ_ben_, МPа	E_ben_, МPа	σ_t_, МPа	E_t_, МPа	a_im_, kJ/m^2^	Shore D	T_v_, °C
100 ED-20	40	2654	26	2040	3	80	86
100 ED-20 + 40 TCEP	53	1750	36	1610	8	72	100
100 ED-20 + 40 TCEP + 0.05 ASM	65	1845	41	1690	10	76	105
100 ED-20 + 40 TCEP + 0.1 ASM	112	2450	58	1842	12	78	127
100 ED-20 + 40 TCEP + 0.5 ASM	100	2558	55	2105	11	80	130
100 ED-20 + 40 TCEP + 10 ASM	75	3450	38	2715	8	82	144
100 ED-20 + 40 TCEP + 30 ASM	80	4690	40	2894	7	83	156
100 ED-20 + 40 TCEP + 50 ASM	88	5211	42	3132	9	84	170
100 ED-20 + 40 TCEP + 60 ASM	65	8339	30	3685	5	85	180
Compositions containing ASMs functionalized with aminoacetic acid							
100 ED-20 + 40 TCEP + 0.1 ASM_(aminoacetic acid)_	130	3270	70	2154	18	83	138
100 ED-20 + 40 TCEP + 50 ASM_(aminoacetic acid)_	112	7168	52	4074	15	88	178

Note: σ_ben_—bending stress; E_ben_—modulus of elasticity in bending; σ_t_—tensile strength; E_t_—modulus of elasticity in tension; a_im_—impact strength; T_v_—Vicat heat resistance; coefficient of variation for properties 3–5%.

**Table 3:** DMA results for epoxy composites.

No.	Composition, Parts by Mass, Cured 15 Parts by Mass, PEPA	E′ at 30 °C, MPa	Tg, °C
1	100 ED-20 + 40 TCEP	5125	95.1
2	100 ED-20 + 40 TCEP + 0.1 ASM	5581	105.8
3	100 ED-20 + 40 TCEP + 0.1 ASM_(aminoacetic acid)_	6441	109.9
4	100 ED-20 + 40 TCEP +50 ASM	7578	109.0
5	100 ED-20 + 40 TCEP + 50 ASM_(aminoacetic acid)_	9235	111.9

**Table 4:** Values of curing indices for epoxy compositions.

Composition, Parts by Mass, Cured with 15 Parts by Mass of PEPA	τ_g_, Min	τ_c_, Min	T_max_, °C	Х, %
100 ED-20 + 40 TCEP	45	53	105	90
100 ED-20 + 40 TCEP + 0.1 ASM	40	58	142	91
100 ED-20 + 40 TCEP + 0.1 ASM_(aminoacetic acid)_	36	54	148	94
100 ED-20 + 40 TCEP + 50ASM	42	73	97	93
100 ED-20 + 40 TCEP + 50 ASM_(aminoacetic acid)_	40	64	103	95

Note: τ_g_—time of gelation; τ_c_—time of curing; T_max_—maximum self-heating temperature; X—curing degree.

**Table 5:** Results of differential scanning calorimetry of epoxy compositions.

Composition, Parts by Mass, Cured with 15 Parts by Mass of PEPA	T_start_–T_end_ T_max_ °C	H, J/g
100 ED-20 + 40 TCEP	53.6–152.6 96.3	484.8
100 ED-20 + 40 TCEP + 0.1 ASM	46.2–159.7 101.8	540.6
100 ED-20 + 40 TCEP + 0.1 ASM_(aminoacetic acid)_	43.0–156.1 98.1	598.2
100 ED-20 + 40 TCEP + 50 ASM	52.2–161.8 102.0	472.7
100 ED-20 + 40 TCEP + 50 ASM_(aminoacetic acid)_	41.6–161.5 98.2	503.7

Note: T_start_, T_end_—temperature of the start and end of the curing process; T_max_—the temperature of the maximum heat release during curing; H—the thermal effect of the reaction.

**Table 6:** The results of the TGA of the epoxy composites.

Composition, Parts by Mass, Cured with 15 Parts by Mass of PEPA	T_5%_, °C	T_30%_, °C	T_40%_, °C	T_50%_, °C	T_60%_, °C	T_70%_, °C	Residues at 900 °C, wt.%
100 ED-20 + 40 TCEP	180	285	310	372	465	527	2.4
100 ED-20 + 40 TCEP + 0.1 ASM	178	292	322	410	482	538	6.9
100 ED-20 + 40 TCEP + 0.1 ASM_(aminoacetic acid)_	184	306	334	415	505	577	11.3
100 ED-20 + 40 TCEP + 50 ASM	190	325	440	495	542	652	27.7
100 ED-20 + 40 TCEP + 50 ASM_(aminoacetic acid)_	204	335	449	530	585	782	29.8

Note: T_5%_, T_30%_, T_40%_, T_50%_, T_60%_, T_70%_—the temperature at a weight loss of 5%, 30%, 40%, 50%, 60%, and 70%, respectively.

**Table 7:** Comparison of developed composites with analogues.

Composition	σ_ben_, МPа	E_ben_, МPа	σ_t_, МPа	E_t_, МPа	a_im_, kJ/m^2^	Shore D
100 ED-20 + 40 TCEP + 0.1 ASM_(aminoacetic acid)_	130	3270	70	2154	18	83
100 ED-20 + 40 TCEP + 50 ASM_(aminoacetic acid)_	112	7168	52	4074	15	88
Analogues						
Epoxy + 40 fly ash (40 wt.%) + nanoclay (3 wt.%) [63]	89.3	-	64.1	-	14.5	-
Epoxy + tamarind shell powder (35 wt.%) + waste marble dust powder (15 wt.%) [64]	38.5	10960	34.6	-	-	-
Epoxy + granite fly dust (20 wt.%) [65]	90	3920	42	2817	-	88
Epoxy + silanized peanut shell (5 wt.%) [66]	63.0	1352	42.7	1618	8.2	-
Epoxy + MnO (2.5 wt.%) [67]	119.1	3133	46.8	3714	4.5	83

Note: σ_ben_—bending stress; E_ben_—modulus of elasticity in bending; σ_t_—tensile strength; E_t_—modulus of elasticity in tension; a_im_—impact strength.

## Data Availability

The data are contained within the article.
